# Fatigue and sleep in children and adolescents with juvenile idiopathic arthritis:a cross-sectional study

**DOI:** 10.3906/sag-1711-167

**Published:** 2019-02-11

**Authors:** Ela TARAKCI, Nilay ARMAN, Kenan BARUT, Sezgin ŞAHİN, Özgür KASAPÇOPUR

**Affiliations:** 1 Department of Neurological Physiotherapy and Rehabilitation, Division of Physiotherapy and Rehabilitation,Faculty of Health Sciences, İstanbul University-Cerrahpaşa, İstanbul Turkey; 2 Department of Physiotherapy and Rehabilitation, Division of Physiotherapy and Rehabilitation,Faculty of Health Sciences, İstanbul University-Cerrahpaşa, İstanbul Turkey; 3 Department of Pediatric Rheumatology, Medical Faculty of Cerrahpaşa, İstanbul University-Cerrahpaşa, İstanbul Turkey

**Keywords:** Juvenile idiopathic arthritis, fatigue, sleep, pain, function

## Abstract

**Background/aim:**

The aims of this study were to primarily investigate fatigue and sleep and to secondarily examine possible relationships between disease activity, pain, and functional ability in children and adolescents with juvenile idiopathic arthritis (JIA).

**Materials and methods:**

Ninety-six patients were enrolled in the study. Disease activity, functional ability, fatigue symptoms, fatigue severity, and sleep quality were assessed with the Juvenile Arthritis Disease Activity Score (JADAS), Childhood Health Assessment Questionnaire (CHAQ), Pediatric Quality of Life Inventory-Multidimensional Fatigue Scale (PedsQL-F), visual analog scale (VAS), and Pittsburgh Sleep Quality Index (PSQI), respectively.

**Results:**

Fatigue severity was moderate to high in 75% of patients with JIA and sleep quality was poor in 40% of them. VAS-fatigue was correlated with VAS-pain, VAS-wellbeing, PSQI, and sleep duration (P < 0.001). Significant relationships were found between the PedsQL-F and all other parameters except JADAS (P < 0.05). VAS-fatigue, CHAQ, and PSQI were identified as significant predictors of PedsQL-F (P < 0.05). Sleep quality, pain, and sleep duration were also significant predictors of fatigue severity (P < 0.05).

**Conclusion:**

This study suggests that fatigue and sleep problems are common problems in JIA. If underlying factors of fatigue and sleep are understood, strategies for improving sleep/fatigue paradox may develop in JIA.

## 1. Introduction 

Juvenile idiopathic arthritis (JIA) is a broad term that describes a clinically heterogeneous group of arthritis of unknown cause, which begins before 16 years of age (1). Children with JIA become fatigued easily; experience joint inflammation, pain, and limited mobility; and report poor sleep quality and daytime sleepiness (2–6). It is reported in the literature that children with JIA suffer from poor sleep, parasomnias, daytime sleepiness, sleep fragmentation, cyclic alternating pattern increase, and sleep-disordered breathing compared to healthy children (2,4). Sleep was disturbed in almost half of the patients with both JIA and juvenile dermatomyositis, and sleep disturbance and fatigue were both correlated with disease activity. Increased pain is associated with more sleep disturbance and more fatigue, and these appear to negatively influence quality of life (3). The cause of sleep disturbance in patients with JIA has yet to be elucidated. Zamir et al. (2) highlighted that the sleep abnormality in JIA patients was associated with pain, while Ward et al. (7) remarked that sleep duration was associated with symptoms of fatigue. Bloom et al. suggested a bidirectional interplay between pain and sleep disturbance (4).

A study reported increased fatigue in children with polyarticular JIA, irrespective of whether they had active or inactive disease, compared to healthy controls (8). Other studies clarified statistically significant correlations between parent-reported fatigue and parent/child-reported disease activity, including parent/child-reported tender and swollen joint counts, and physician assessment of disease activity (3,9).

In another study, it was reported that functional ability was most strongly associated with parent- and child-reported fatigue and pain was underlined as an important confounder between disease activity and fatigue (10). Similarly, another study demonstrated a significant association between fatigue and functional ability in adult patients with rheumatoid arthritis (11). In another study, it was remarked that children and adolescents with JIA suffer from chronic or recurrent pain and disability, which can severely limit their ability to complete daily physical tasks and participate in school and social activities (12). However, relations among sleep quality, pain, fatigue, and functional ability in children with JIA have been not been well studied. The aims of this study were to primarily investigate fatigue and sleep and to secondarily examine possible relationships between disease activity, pain, and functional ability in children and adolescents with JIA.

## 2. Materials and methods

### 2.1. Patients

All patients attending the outpatient clinic of the Department of Pediatric Rheumatology were recruited between February 2015 and December 2015 to participate in this study. All participants signed informed consent forms. The participants were volunteers who were examined by the pediatric rheumatologist who participated in the present study. Approval for this study was obtained from the Research Ethics Committee of İstanbul University (No: A29/03.Feb.2015) and the study was conducted in accordance with the Declaration of Helsinki.

Inclusion criteria consisted of a diagnosis of JIA according to the International League of Associations for Rheumatology criteria (13), being aged between 6 and 18 years, and being able to read and write in Turkish. All patients were diagnosed with JIA 6 months prior to the study. Patients were excluded from the study if they had a second rheumatic or other chronic disease, a history of mental deficit or psychological problems, or no approval from their families to participate in the study. When all patients attending our clinic during the study duration were assessed, 96 of them fulfilled the inclusion criteria and had approval from their families to participate. Thus, the sample size was 96 patients. These patients’ characteristics (sex and age) and disease characteristics (disease duration and disease activity) were collected by the physiotherapists. 

### 2.2. Evaluation of disease activity

A blood sample was taken for erythrocyte sedimentation rate (ESR) and disease activity was assessed by the pediatric rheumatologist using the Juvenile Arthritis Disease Activity Score (JADAS). JADAS is a new composite disease activity score speciﬁc to JIA. It is simple to calculate using four variables measured in the clinical setting: active joint count, physician global, parents global, and ESR. JADAS has three practicable versions, which differ in the active joints count incorporated: JADAS10, JADAS27, and JADAS71. JADAS27 includes the following joints: cervical spine, elbows, wrists, metacarpophalangeal joints (from first to third), proximal interphalangeal joints, hips, knees, and ankles. In this study, JADAS27 was preferred for its better performance in validation analyses (14). JADAS27 was calculated as the simple sum of the scores of its four components, which yields a global score of 0–57 (27 joints + physician global with 10-cm visual analog scale (VAS) + parent global with 10-cm VAS + ESR). The ESR value is normalized to a 0–10 scale according to the following formula: (ESR (mm/hour) – 20)/10. Before making the calculation, ESR values of <20 mm/h were converted to 20 and ESR values of >120 m/h were converted to 120 (15). The JADAS27 cutoff score for low disease activity was ≤2.7 and for high disease activity was ≥6 (16).

### 2.3. Evaluation of functional ability

Functional ability was assessed using the Turkish version of the Childhood Health Assessment Questionnaire (CHAQ). CHAQ is a reliable and valid tool for the assessment of functional ability in patients with JIA (17). In 8 activities (dressing/grooming, arising, eating, walking, hygiene, reach, grip, and activities), a number of questions were answered and scored on a scale of 0–3, where 0 = able to do with no difficulty, 1 = able to do with some difficulty, 2 = able to do with much difficulty, and 3 = unable to do. The mean of the 8 scores identified the CHAQ score (range: 0–3). CHAQ also provided an assessment of discomfort using a 100-mm VAS for the evaluation of pain and a 100-mm VAS for the evaluation of overall wellbeing. A score of 0 indicated “no pain” and 100 indicated “extreme pain” for VAS pain, and a score of 0 indicated “very well” and 100 “very bad” for VAS overall wellbeing. A higher score indicated more problems in both pain and overall wellbeing (18). 

### 2.4. Evaluation of fatigue

Fatigue was evaluated in detail as fatigue symptoms and fatigue severity. Fatigue symptoms were evaluated with Pediatric Quality of Life Inventory-Multidimensional Fatigue Scale (PedsQL-F). PedsQL-F is a valid and reliable tool, and it can be used to measure symptom-specific fatigue among patients with JIA (19). PedsQL-F, which is an 18-item questionnaire designed to measure the child’s and the parent’s perception of fatigue in pediatric patients, comprises a general fatigue scale (6 items), sleep/rest fatigue scale (6 items), and cognitive fatigue scale (6 items). Fatigue severity was assessed with a 100-mm VAS. A VAS score of <20 mm indicated “low fatigue”, VAS score between 20 and 49 mm indicated “clinically relevant fatigue”, and VAS score of ≥50 mm indicated “high fatigue” (20).

### 2.5. Evaluation of sleep

Sleep quality was evaluated with the Pittsburgh Sleep Quality Index (PSQI). The PSQI is a 23-item questionnaire that generates scales reflecting daytime dysfunction, sleep latency, disturbance, duration, quality, and efficiency. As the score increases, sleep quality decreases and daytime dysfunction due to sleep quality disorder increases. The PSQI is composed of three main scores: total PSQI score, PSQI subscores, and sleep quality status score. PSQI subscores are subjective sleep quality, time needed to fall into sleep, sleep efficiency, use of medication, and daytime sleepiness. Each component is scored from 0 to 3, leading to a global PSQI score between 0 and 21, with higher scores indicating lower quality of sleep (21). The PSQI is useful to identify good and poor sleepers. A global PSQI score above 5 indicates poor sleep (22). Patients were also asked about times of falling asleep and getting up. Sleep time was calculated by asking the patients about their sleeping and awakening hours.

### 2.6. Statistical analysis

Data were evaluated using SPSS 21.0 for Windows (IBM Corp., Armonk, NY, USA) and by analyzing descriptive statistics (frequency, mean, and standard deviation). Before the statistical analysis, the Kolmogorov–Smirnov test was used to test for normal distribution of data. The independent sample t-test was used to determine differences of subjects’ outcome scores because the data were normally distributed. Intercorrelations between parameters were computed with Pearson’s correlation analysis. Variables that were significantly associated with PedsQL-F-patient and VAS-fatigue by Pearson’s correlation analysis were included in the multivariable model. Stepwise selection using a significance level of P < 0.05 was employed to obtain the final multivariable models. P < 0.05 was considered statistically significant for all tests. Bonferroni’s correction was applied (P < 0.05/n, where n = number of groups) when multiple comparisons were made and P < 0.017 was considered statistically significant for fatigue severity groups.

## 3. Results

### 3.1. Patients characteristics 

A total of 96 eligible patients with JIA (61 female, 35 male) were enrolled including 42 (43.8%) with polyarticular onset (poly-JIA), 33 (34.8%) with oligoarticular (oligo-JIA) subtype, 14 (14.6%) with systemic onset (ss-JIA), and 7 (7.3%) with other subtypes. The clinical characteristics and scores of all outcomes of the patients are presented in Table 1. 

**Table 1:** Demographic and clinical results of patients with juvenile idiopathic arthritis.

Demographic and clinical results	Patients (n = 96) Mean ± SD / Median (IQR)
Age (years)	12.93 ± 3.36
BMI (kg/m2)	19.16 ± 3.89
Disease duration (years)	5.78 ± 3.75
JADAS	3.75 (5.13)
PedsQL-F	67.62 ± 22.40
VAS-fatigue	40 (30)
VAS-pain	17.50 (40)
VAS-wellbeing	30 (40)
CHAQ	0.37 (0.87)
PSQI	4 (3)
Sleep duration (h/day)	8.43 ± 1.59
	n (%)
Disease activity (low/moderate/high)	44/32/20 (45.9%, 33.3%, 20.8%)
Sex (F/M)	61/35 (63.5%, 36.5%)

JIA: Juvenile idiopathic arthritis, BMI: body mass index, F: female, M: male, JADAS: Juvenile Arthritis Disease Activity Score, VAS: visual analog scale, PedsQL-F: Pediatric Quality of Life Inventory-Multidimensional Fatigue Scale, CHAQ: Childhood Health Assessment Questionnaire, PSQI: Pittsburgh Sleep Quality Index.

### 3.2. Fatigue

According to the VAS-fatigue data, only 25% of the patients (n = 24) reported having a low level of fatigue (<20 mm); the VAS-fatigue score was between 20 and 49 mm for 44.8% of the patients (n = 43) having clinically relevant fatigue and ≥50 mm (high fatigue) for 30.2% (n = 29). Table 2 shows scores of JADAS, PedsQL-F, VAS-pain, VAS-wellbeing, CHAQ, PSQI, and sleep duration in the fatigue severity groups. There, ≥50 mm was statistically significant different in the comparisons of the results of JADAS, PedsQL-F, VAS-pain, VAS-wellbeing, PSQI, and sleep duration in the fatigue severity groups. In particular, the differences between patients with low levels of fatigue and high levels of fatigue were statistically significant (P < 0.017).

**Table 2:** Disease activity, fatigue symptoms, pain severity, wellbeing, functional ability, sleep quality, and sleep duration in the fatigue severity groups.

	VAS-F < 20 mm(1) n = 24 Median (IQR)	VAS-F of 20–49 mm(2) n = 43 Median (IQR)	VAS-F ≥ 50 mm(3) n = 29 Median (IQR)	P			
				(1)-(2)-(3)	(1)-(2)	(1)-(3)	(2)-(3)
JADAS	1.60 (2)	3 (5.10)	4 (3.90)	0.041	0.286	0.025	0.329
PedsQL-F	91.66 (20.83)	73.61 (20.83)	58.33 (23.25)	0.003	0.550	0.004	0.022
VAS-pain	0 (5)	20 (40)	40 (47.75)	0.0001	0.209	0.000	0.007
VAS-wellbeing	10 (25)	30 (30)	50 (23.8)	0.001	0.019	0.001	0.321
CHAQ	0.25 (0.75)	0.25 (0.37)	0.56 (1)	0.535	0.582	0.983	0.669
PSQI	2 (2)	4 (2)	5 (4)	0.002	0.262	0.001	0.039
Sleep Duration (h/day)	9 (2)	8 (3)	8 (2)	0.010	0.356	0.008	0.100

VAS-F: Visual analog scale-fatigue. (1): VAS-F < 20 mm. (2): VAS-F of 20–49 mm. (3): VAS-F ≥ 50 mm. JADAS: Juvenile Arthritis Disease Activity Score. PedsQL-F: Pediatric Quality of Life Inventory-Multidimensional Fatigue Scale. CHAQ: Childhood Health Assessment Questionnaire. PSQI: Pittsburgh Sleep Quality Index. Bonferroni’s correction, P < 0.01.

When the JIA subtypes were compared due to scores for PedsQL-F (poly-JIA = 67.92 ± 21.02, oligo-JIA = 67.63 ± 23.54, ss-JIA = 64.58 ± 28.26, other = 71.82 ± 13.90) and VAS-fatigue (poly-JIA = 3.64 ± 1.93, oligo-JIA = 3.59 ± 2.82, ss-JIA = 2.69 ± 2.21, other = 4.28 ± 1.88), there was no statistically significant difference in the comparisons of the results of PedsQL-F (P = 0.919) or VAS-fatigue (P = 0.473) in the JIA subtype groups. 

### 3.3. Sleep

The mean of sleep duration was 8.43 ± 1.59 h, while 66 (68.8%) patients reported that they slept less than 10 h. Fifty-eight (60.4%) patients had PSQI scores of <5, indicating good sleep quality, while 38 (39.6%) patients had PSQI scores of >5, indicating poor sleep quality. When the JIA subtypes were compared according to PSQI scores (poly-JIA = 4.74 ± 2.26, oligo-JIA = 3.79 ± 2.58, ss-JIA = 3.5 ± 2.24, other = 5 ± 1.91) and sleep duration (poly-JIA = 8.25 ± 1.52, oligo-JIA = 8.79 ± 1.67, ss-JIA = 8.42 ± 1.28, other = 7.85 ± 2.11), there was no statistically significant difference in the comparisons of the results of the PSQI (P = 0.166) or sleep duration (P = 0.377) in the JIA subtype groups. PSQI scores were not different due to JIA subtype. Table 3 shows the comparisons of the results of sleep duration, JADAS, PedsQL-F, VAS-fatigue, CHAQ, VAS-pain, and VAS-wellbeing in patients with good/poor sleep quality. Almost all parameters of patients with poor sleep were statistically significantly worse than in patients with good sleep (P < 0.05).

**Table 3:** Comparisons of the results of disease activity, fatigue symptoms, fatigue severity, pain severity, wellbeing, functional ability, and sleep duration in patients in good/poor sleep quality groups.

	Good sleepn = 58 (60.4%)	Poor sleepn = 38 (39.6%)		
	Mean ± SD	Mean ± SD	t	p
Sleep duration (h)	8.72 ± 1.52	7.98 ± 1.61	2.242	0.028
JADAS	4.65 ± 4.31	6.96 ± 4.96	–2.418	0.018
PedsQL-F	75.45 ± 19.19	55.66 ± 21.86	4.674	0.000
VAS-fatigue	28.4 ± 22.8	49.7 ± 19.9	–4.508	0.000
VAS-pain	16.81 ± 22.25	34.47 ± 25.00	–3.620	0.000
VAS-wellbeing	27.06 ± 24.13	53.15 ± 22.94	–5.281	0.000
CHAQ	0.49 ± 0.53	0.68 ± 0.70	–1.528	0.130

JADAS: Juvenile Arthritis Disease Activity Score. VAS: Visual analog scale. PedsQL-F: Pediatric Quality of Life Inventory-Multidimensional Fatigue Scale. CHAQ: Childhood Health Assessment Questionnaire.

### 3.4. Relationships between disease activity, fatigue, functional ability, pain, wellbeing, sleep quality, and sleep duration

Table 4 shows the correlations of the results of JADAS, PedsQL-F, CHAQ, VAS-fatigue, VAS-pain, VAS-wellbeing, PSQI, and sleep duration. Significant relationships were found between JADAS and VAS-fatigue (r = 0.303), VAS-pain (r = –0.329), and VAS-wellbeing (r = –0.330) (P < 0.05). Significant relationships were also found between PedsQL-F and all other parameters except JADAS (P < 0.05). VAS-fatigue was significantly correlated with VAS-pain (r = 0.544), VAS-wellbeing (r = 0.539), PSQI (r = 0.496), and sleep duration (r = –0.229) (P < 0.001). On the other hand, it was found that CHAQ was significantly correlated with PedsQL-F (r = –0.501), VAS pain (r = 0.298), and VAS-overall wellbeing (r = 0.331); VAS-pain was significantly correlated with VAS-overall wellbeing (r = 0.583) and PSQI (r = 0.328); and VAS-wellbeing was significantly correlated with PSQI (r = 0.471) (P < 0.001). In addition, sleep duration was significantly correlated with PSQI (r = –0.272) (P < 0.01). It was also found that age (r = –0.091, P = 0.378), sex (r = 0.024, P = 0.815), disease duration (r = 0.003, P = 0.981), and disease type (r = 0.008, P = 0.936) were not statistically correlated with PedsQL-F. Similarly, it was found that age (r = –0.106, P = 0.303), sex (r = –0.17, P = 0.314), disease duration (r = 0.140, P = 0.185), and disease type (r = –0.012, P = 0.908) were not statistically correlated with VAS-fatigue.

**Table 4:** Correlations of the results of disease activity, fatigue symptoms, fatigue severity, pain severity, wellbeing, functional ability, sleep quality, and sleep duration in patients with juvenile idiopathic arthritis

	JADAS	PedsQL-F	VAS-fatigue	CHAQ	VAS-pain	VAS-global wellbeing	PSQI
PedsQL-F	r = 0.030P = 0.771						
VAS-fatigue	r = 0.303P = 0.003	r = –0.484P = 0.0001					
CHAQ	r = 0.179P = 0.586	r = –0.501P = 0.0001	r = 0.124P = 0.402				
VAS-pain	r = –0.329P = 0.011	r = –0.329P = 0.001	r = 0.544P = 0.0001	r = 0.298P = 0.023			
VAS-wellbeing	r = 0.330P = 0.001	r = –0.357P = 0.001	r = 0.539P = 0.0001	r = 0.331P = 0.002	r = 0.583P = 0.0001		
PSQI	r = 0.182P = 0.076	r = –0.492P = 0.0001	r = 0.496P = 0.0001	r = 0.184P = 0.380	r = 0.328P = 0.001	r = 0.471P = 0.001	
Sleep duration	r = 0.087P = 0.411	r = 0.242P = 0.018	r = –0.229P = 0.001	r = 0.032P = 0.986	r = –0.129P = 0.133	r = –0.143P = 0.141	r = –0.272P = 0.008

JADAS: Juvenile Arthritis Disease Activity Score. VAS: Visual analog scale. PedsQL-F: Pediatric Quality of Life Inventory- Multidimensional Fatigue Scale. CHAQ: Childhood Health Assessment Questionnaire. PSQI: Pittsburgh Sleep Quality Index.

### 3.5. Predictors of fatigue

In multivariate linear regression analysis, VAS-fatigue, CHAQ, and PSQI were identified as statistically significant predictors of PedsQL-F (P < 0.05). Moreover, sleep quality, pain, and sleep duration were statistically significant factors of fatigue severity (P < 0.05) (Table 5). In addition, the clinical features of age, sex, disease duration, and disease type were not significant predictors of PedsQL-F or VAS-fatigue (P > 0.05). 

**Table 5:** Multivariate linear regression analysis of fatigue symptoms and fatigue severity in patients with juvenile idiopathic arthritis.

Predictor variable		PedsQL-F						VAS-fatigue				
	B	Std.error	%95 CI	Beta	t	P	B	Std. error	95% CI	Beta	t	P
Intercept	86.92	11.13	64.78 to 109.06		7.80	0.0001	3.45	1.15	1.16 to 5.74		2.99	0.004
PSQI	–2.15	0.87	–3.91 to –0.35	–0.23	–2.38	0.019	0.32	0.08	0.13 to 0.48	0.30	3.52	0.001
VAS-pain	0.05	0.09	–0.13 to 0.24	0.06	0.62	0.535	0.03	0.01	0.008 to 0.04	0.28	2.87	0.005
VAS-global wellbeing	0.05	0.08	–0.10 to 0.24	0.08	0.82	0.411	0.01	0.009	–0.005 to 0.03	0.14	1.45	0.150
Sleep duration	0.68	1.13	–1.25 to 3.28	0.07	0.88	0.378	–0.28	0.11	–0.55 to –0.08	–0.21	–2.71	0.008
CHAQ	–16.39	2.99	–22.02 to –9.92	–0.45	–5.25	0.000	–0.31	0.32	–0.49 to 1.16	0.08	0.80	0.422
JADAS	1.05	0.47	–0.93 to 1.34	0.03	0.35	0.722	0.08	0.05	–0.01 to 0.18	0.13	1.62	0.108
VAS-fatigue	–3.93	1.01	–5.39 to –1.34	–0.37	–3.30	0.001						

Std: Standard. CI: Confidence interval. JADAS: Juvenile Arthritis Disease Activity Score. VAS: Visual analog scale. PedsQL-F: Pediatric Quality of Life Inventory-Multidimensional Fatigue Scale. CHAQ: Childhood Health Assessment Questionnaire. PSQI: Pittsburgh Sleep Quality Index.

## 4. Discussion

We found that fatigue affects 75% of patients with JIA, thus making fatigue a common problem among this patient population. We also found that 40% of them have poor sleep quality and 69% of the patients slept less than 10 h. When considering the percentile curves of sleep duration presented by Iglowstein et al. (23), sleep durations of the patients were less than those of healthy children. In line with other studies (7,24–26), we found that fatigue and sleep problems were also common problems in patients with JIA. According to the results of our study, sleep quality, sleep duration, and pain were predictors for fatigue severity. Furthermore, fatigue severity, sleep quality, and functional ability were predictors for fatigue symptoms. Another important result of our study was that disease activity was not a predictor of fatigue severity, whereas a significant correlation was found between disease activity and fatigue severity.

Our study has an important strong aspect. Unlike most studies of fatigue in patients with JIA, we also assessed fatigue symptoms and fatigue severity. We measured fatigue symptoms using a validated and disease-specific questionnaire that is commonly used in studying patients with JIA (19). Besides, we have used the most common scale to measure fatigue severity because we considered that fatigue symptoms and fatigue severity may be associated with different factors. 

Previous studies have shown diverging results regarding the relationships among fatigue, pain, functional ability, and disease activity (3,8,10,27,28). Actually, none of these studies evaluated fatigue in JIA multidimensionally. Some of them found that fatigue was associated with disease activity (10,27,29), but some of them reported that fatigue was not associated with disease activity (3,7). Our finding is the same as the last finding that disease activity did not have an independent effect on fatigue, although 45.8% of our patients with JIA had low disease activity. This difference in outcomes may be attributed to differences in several factors, including patient age, the questionnaires used, and the treatment setting. On the other hand, pain is clinically the most common symptom in patients with JIA (30,31). Our finding showed that pain was a fairly strong predictor of fatigue severity, while pain was correlated with fatigue symptoms and severity in patients with JIA. Similarly, previous studies reported that a significant positive correlation was found between fatigue and pain in patients with JIA (3,7,8). As a consequence, pain affects other problems in patients with JIA because of its biopsychosocial dimension. Our results also suggested that functional ability was the strongest predictor of fatigue symptoms, although it was not correlated with fatigue severity in patients with JIA. Similarly, Rignold et al. (10) noticed that functional ability, as measured by CHAQ, was most strongly associated with fatigue in patients with JIA. Bromberg et al. (28) noticed that fatigue was not a predictor for functional limitations despite high levels of fatigue in patients with JIA. This contradiction in results may be due to differences between measurement methods of fatigue. Moreover, the relationship between fatigue and functional ability has rarely been studied in the literature. Eventually, our finding emphasized that fatigue severity and symptoms were affected by different factors. Pain, sleep duration, and sleep quality were predictors of fatigue severity, while functional ability, sleep quality, and fatigue severity were predictors of fatigue symptoms in patients with JIA. Our results will contribute to clarifying the underlying reason for this dilemma.

Sleep is a critical developmental need for children and adolescents and, when disturbed, is associated with impairments in daily activities (32). Few studies have examined sleep in children with JIA. In one study, sleep duration was associated with fatigue symptoms (7). Laplant et al. (33) similarly showed that fatigue symptoms were related to insomnia. In our study, sleep duration was the strongest predictor of fatigue severity. Additionally, while sleep duration of patients with JIA was decreased, fatigue severity was significantly increased. Patients with high fatigue severity had significantly more pain severity than patients with low fatigue. Several previous studies also demonstrated an association between poor sleep quality and chronic widespread pain (2,3,6,34). Similarly, we found that sleep quality was associated with pain and also with fatigue symptoms and severity. It appears reasonable that increased pain may have caused poor sleep in our subjects. It is difficult to ascertain whether disturbed sleep causes increased pain and fatigue, or whether pain and increased fatigue lead to poor sleep. We believe that the relationship between pain, poor sleep, and fatigue is like a paradox in patients with JIA. Similarly, Butbut et al. (3) supposed that this relationship may be a vicious cycle with a significant influence on quality of life. Lewin and Dahl (35) verified that a bidirectional theory of sleep disturbance and pain can be suggested: not only can pain interfere with sleep, but insufficient sleep can also have negative effects on pain management. This cyclic pattern could produce increased attention to pain problems, affective disruption, and high-risk behaviors, all of which can intensify the pain experience and accordingly disturb the sleep. Breaking this vicious cycle may be an appropriate therapeutic target for improving quality of life in patients with JIA. As in our results, patients with good sleep have a higher wellbeing. Thus, sleep quality is important to break this vicious cycle.

There were several limitations in this study. Previous studies indicated that psychological and emotional states affect fatigue and sleep in patients with JIA, but we did not assess the psychological and emotional states of our patients. Also, we did not compare our patients with a healthy control population, as we observed that the differences in sleep and fatigue between JIA patients and healthy controls had been adequately demonstrated.

In conclusion, fatigue and sleep disturbance are considerable problems in patients with JIA. If underlying factors of fatigue and sleep are understood, strategies for improving the sleep/fatigue paradox may be developed in patients with JIA. Moreover, these strategies should entail a holistic and biopsychosocial approach. Therefore, further studies should focus on all factors that may affect fatigue and sleep comprehensively and investigate the effects of these factors on the treatment of patients with JIA. 

## Acknowledgments

The authors thank all patients and parents who helped this study in the Department of Pediatric Rheumatology, Medical Faculty of İstanbul University-Cerrahpaşa, and also Farzin Haj Ebrahimi for editing the English.
